# Compromised osteogenic effect of exosomes internalized by senescent bone marrow stem cells *via* endocytoses involving clathrin, macropinocytosis and caveolae

**DOI:** 10.3389/fbioe.2022.1090914

**Published:** 2023-01-04

**Authors:** Lei Qi, Weiwen Ge, Cancan Pan, Weidong Jiang, Dan Lin, Lei Zhang

**Affiliations:** ^1^ Department of Oral and Cranio-Maxillofacial Surgery, Shanghai Ninth People’s Hospital, College of Stomatology, Shanghai Jiao Tong University School of Medicine, National Clinical Research Center for Oral Diseases, Shanghai Key Laboratory of Stomatology and Shanghai Research Institute of Stomatology, Shanghai, China; ^2^ Shanghai University of Medicine and Health Sciences, Shanghai, China

**Keywords:** exosomes, bone marrow stem cells, senescence, bone regeneration, endocytosis, osteogenic differentiation

## Abstract

Stem cell senescence leads to progressive functional declines and disrupts the physiological homeostasis of bone environment. Stem cell-derived exosomes are emerging as promising therapeutical approaches to treat diverse aging-related osseous diseases. Herein, a previously reported osteoinductive exosome (OI-exo) was applied as a therapeutic agent for bone repair in aging individuals and its internalization mechanisms in senescent bone marrow stem cells (BMSCs) were explored. The results demonstrated that OI-exos derived from young BMSCs could partially rescue the proliferation, osteogenic differentiation and alleviate aging phenotypes *in vitro*. OI-exo-delivered hierarchical mesoporous bioactive glass (MBG) scaffold effectively promote *in vivo* bone formation in aging rat cranial defect model. However, the osteogenic effects of OI-exo both *in vitro* and *in vivo* were compromised in senescent individuals and for aging BMSCs compared to younger ones. This study revealed that non-senescent BMSCs internalized exosomes exclusively *via* clathrin-mediated endocytosis, while senescent BMSCs additionally evoked macropinocytosis and caveolae-mediated endocytosis to mediate the internalization of exosomes. The alteration of endocytic manner of senescent BMSCs and the involvement of macropinocytosis might be responsible for the compromised effects of therapeutical exosomes. The phenomena discovered in this study could also be extended to other scenarios where drugs or treatments exerted compromised effects in aging individuals. The influence of endocytic manner, avoidance of macropinocytosis-related negative effects should be taken into considerations in future therapeutic design for aging populations.

## 1 Introduction

Bone, as the fundamental part of motor system that supports and protects the human body, continuously undergoes self-renewal process throughout life, realized *via* the balance between resorption by osteoclasts and remineralization by osteoblasts (Wang et al., 2020; Li, Q, et al., 2022; Cao et al., 2018). Senescence at the organism or cellular level can disrupt the bone remodeling balance where decreased osteogenesis failed to compensate bone resorption, ultimately resulting in osteoporosis, a common chronic disease in aging people with high complication rate of bone fractures (Paschalis et al., 2016; Yu and Wang 2016). At the cellular level, the main manifestation of bone aging is a continuous decline in metabolic activity of bone marrow stem cells (BMSCs) (Li, X, et al., 2022), which, as the population of pluripotent MSCs, can differentiate into osteoblasts and eventually osteocytes as well as other functioning cells (Cancedda, Giannoni, and Mastrogiacomo 2007) BMSCs senescence, like other cells, is an irreversible process that causes cell cycle arrest and cellular phenotypic alterations including changes in gene expression, DNA damage, telomere shortening as well as development of complex pro-inflammatory pathways (Peng et al., 2020; G; Li et al., 2021; C; Li et al., 2017). Although the mechanisms that cause these senescent changes are not completely understood, increasing evidences have demonstrated that the accumulative age-related molecular and cellular damage of BMSCs include genomic instability, epigenetic alterations, mitochondrial dysfunction, and altered intercellular communication that cause functional vandalization in bone tissue (Zhao, Y et al., 2022; Cai et al., 2022; Yang et al., 2017).

Rejuvenation or functional restoration of senescent BMSCs have emerged as a novel therapeutic approach for preventing and treating skeletal aging, including treatments of traditional Chinese medicine, chemical drugs, and nucleic acid drugs (Kang et al., 2022; Yi et al., 2021; L; Hu et al., 2022). Exosomes, the intercellular communicational vesicles (30–100 nm) that contain functional nucleic acids including DNAs, mRNAs and miRNAs (Tkach and Théry 2016; Valadi et al., 2007; O'Brien et al., 2020; Liao et al., 2021), have been explored for therapeutical applications in recent years. As natural secretion of host cells, exosomes are incorporated into recipient cells for information exchange without immunogenicity, and deliver rich contents in various physiological and pathological conditions including the aging process (X. Liu et al., 2022). For example, exosomal miR-31a-5p has been demonstrated as a key modulator in the age-related bone marrow microenvironment by influencing osteoblastic and osteoclastic differentiation (R. Xu et al., 2018). In our previous study, optimized BMSC-derived osteoinductive exosomes were immobilized in hierarchical MBG scaffolds and demonstrated to efficiently improve osteogenesis through a Bmpr2/Acvr2b competitive receptor-activated Smad pathway (A. Liu et al., 2021).

Many researches focus on the function of the exosomal cargoes in pathological and physiological processes. However, regarding to the different effects of a same treatment on older and younger individuals, the uptake pathways might affect the downstream signaling conduction in the exosome-mediated progression. Endocytosis, as the main mechanism of exosome uptake, is reported to play key roles in the regulation of many intracellular signaling cascades (Gurung et al., 2021). Endocytic pathways including caveolae-dependent, clathrin-dependent, and macropinocytosis are reported as exosome uptake mechanisms of different types of cells, and endocytosis-related molecule expression level influences the uptake capacity of exosomes (Abels and Breakefield 2016; Costa Verdera et al., 2017; Horibe et al., 2018; Joshi et al., 2020). The aging microenvironment alters the morphology and function of cells, especially stem cells, which further influences molecular cell biology of distinct exosomal endocytic mechanisms (McKelvey et al., 2015). However, the different exosome uptake mechanisms between old and young BMSCs remains unidentified.

Herein, the aim of this study was to explore the potential efficacy of previously reported osteoinductive exosome (OI-exo) as a therapeutic agent for bone repair in an aging condition and to investigate its internalization mechanism into old BMSCs in comparison with younger BMSCs. Old BMSCs (O-BMSCs) and young BMSCs (Y-BMSCs) were used to investigate the underlying cellular uptake mechanism and to better understand bone regenerative microenvironment in aging conditions. In addition, OI-exo-delivered hierarchical MBG scaffolds were applied for bone regeneration in rat cranial defect model to explore the *in vivo* therapeutic difference between old and young individuals*.*

## 2 Materials and methods

### 2.1 BMSCs extraction, culture and identification

All animal experiments were performed in compliance with the guidelines developed by the Institutional Animal Care and Use Committees of Shanghai Ninth People’s Hospital, and the protocols were reviewed and approved by the Ethic Committee of Shanghai Ninth People’s Hospital.

Y-BMSCs of 4-week young rats and O-BMSCs of 18-month aged rats were harvested from the femur and tibia as previously described (L. Liu et al., 2019). Briefly, bone marrow was flushed out with α-MEM by a sterile needle, suspended in a-MEM supplemented with 10% fetal bovine serum (FBS; Gibco^®^, Thermo Fisher Scientific Inc. MA, United States) and 1% penicillin/streptomycin, and culture at 37°C in a humidified atmosphere with 5% CO_2_. The BMSCs were passaged when they reached approximately 80%–90% confluence. Passage 3-5 of the BMSCs were used for *in vitro* study. The stemness markers of extracted cells were identified by flow cytometry. Briefly, the cells were collected by enzymatic digestion, washed and resuspended with PBS, and stained with anti-CD29, anti-CD45, anti-CD90 antibodies (Abcam, Cambridge, United Kingdom) on ice for 30 min. The three markers were widely accepted as criteria defining stem cells (Y. Hu et al., 2020). Then, each group of the cells were analyzed by a flow cytometer (FACSCalibur™, Becton Dickinson, NJ, United States).

### 2.2 Osteoinductive exosome (OI-exo) isolation and characterization

Passage 3–5 Y-BMSCs were used for exosome isolation as described in previous study (Luo et al., 2022). Briefly, cells were cultured in an osteogenic medium supplemented with 100 nM dexamethasone, 10 mM *ß*-sodium glycerophosphate and 0.05 mM ascorbic acid for 48 h. Then the supernatant was harvested, 0.22 μm-filtered, centrifuged at 10,000 g for 30 min, then 100,000 g for 90 min. The exosome pellet was collected and resuspended in PBS. The obtained exosomes were stored at −80°C for further use.

For exosome identification, the morphology of OI-exo was observed using transmission electron microscope (TEM; Hitachi H-7650, Japan). The particle size and concentration of the exosome were analyzed using nanoparticle tracking analysis (NTA). The exosomal markers were detected with TSG101, Alix and CD9 antibodies (Abcam, Cambridge, United Kingdom) by western blot.

### 2.3 Characterization of exosome internalization

Exosome were labeled with a red fluorescent dye (Dil, Beyotime) according to the manufacturer’s instructions. The labeled OI-exo were then added to cells (O-BMSCs and Y-BMSCs) and co-cultured for 1, 3, 6, and 12 h. At each time point, the cells were washed with PBS and fixed in 4% paraformaldehyde for 15 min. Nuclei and cytoskeleton were stained with DAPI and FITC-phalloidin, respectively, and observed with confocal laser-scanning microscopy (CLSM; Leica, Germany). The fluorescent intensity of the internalized DiI-labeled OI-Exo was quantified by ImageJ.

To explore the exosome internalization pathways, the cells were incubated with Dil-labeled OI-exo for 3 h. During incubation period, alexa Fluor 488–labeled endocytic markers including transferrin (a marker for clathrin-mediated endocytosis), dextran (a marker for macropinocytosis) and choleratoxin (a marker for caveolae-mediated endocytosis) were added for counterstaining. The cells were fixed, stained with DAPI and observed CLSM. Colocalization of the Dil-labeled OI-exo and alexa Fluor 488–labeled endocytic markers was quantified by the Pearson correlation coefficient and Manders overlap coefficient using ImageJ.

Inhibitors were applied to further verify the exosome internalization pathways. Cells were pre-treated with dynasore (an inhibitor of clathrin-mediated endocytosis), AML (inhibitor of macropinocytosis), cytochalasin D (inhibitor of macropinocytosis), and Methyl-β-cyclodextrin (an inhibitor of caveolae-mediated endocytosis) for 1 h before incubated with Dil-labeled OI-exo for 3 h. The cells were stained with FITC-phalloidin and DAPI, and observed by CLSM. The fluorescent intensity of the internalized DiI-labeled OI-Exo was quantified by Integrated Density/Area using ImageJ.

### 2.4 Evaluation of cell viability, senility and osteogenic differentiation

#### 2.4.1 CCK-8 assay

Proliferation of Y-BMSCs and O-BMSCs cultured with/without OI-exo was quantified by cell counting kit-8 assay (CCK-8; Dojindo, Japan). Briefly, cells were seeded in 96-well plates at a density of 3*10^3^ cells/well and treated with/without OI-exo. After culturing for 1, 3 and 5 days, the cells were incubated with 10 μL CCK-8 solution for 3 h and the absorbance at 450 nm was measured using a microplate reader (BioTek Epoch, United States).

### 2.4.2 SA- *ß*-galactosidase staining

Senescence associated *ß*-galactosidase (SA-β-Gal) staining of Y-BMSCs and O-BMSCs cultured with/without OI-exo was performed using the senescence-associated *ß*-galactosidase staining kit (Beyotime, Jiangsu, China) according to the manufacturer’s instructions. After cell confluence, cells were fixed for 15 min and incubated overnight at 37°C with *ß*-Gal Staining Solution, then ice-cold PBS was used to quench the reaction. The staining was observed in bright field using an inverted optical microscope (Leica DMI6000B, Germany).

#### 2.4.3 ALP staining

Osteogenic differentiation of Y-BMSCs and O-BMSCs cultured with/without OI-exo was evaluated using ALP staining. Cells were seeded in 6-well plates at a density of 1*10^5^ cells/well with/without addition of OI-exo. After culturing for 7 days, the cells were fixed with 4% paraformaldehyde and stained using a BCIP/NBT alkaline phosphatase color development kit (Beyotime, Jiangsu, China) according to the manufacturer’s protocol. Inverted light microscope (Leica DMI6000B, Germany) was applied to observe ALP staining.

#### 2.4.4 Real time qPCR analysis

Osteogenic and senescence-associated gene expressions of Y-BMSCs and O-BMSCs cultured with/without OI-exo were measured by real time qPCR. Briefly, BMSCs were seeded in a 24-well plate at a density of 1*10^5^ cells/well with/without addition of OI-exo. After incubation for 3 days, total RNA was extracted from cells using Trizol reagent (Takara Bio, Japan) according to the manufacturer’s instructions. Reverse transcription was performed with 1,000 ng of total RNA in a final volume of 20 μl using PrimeScript RT reagent kit (Takara Bio, Japan) according to the manufacturer’s recommendations. Then diluted cDNA was mixed with TB Green™ Premix Ex Taq™ (Takara, Tokyo, Japan), forward and reverse primers and RNase free water to perform RT-qPCR. The relative expression level was calculated using the 2^-(△△CT)^ method and normalized to the level of the housekeeping gene *ß*-actin. The primer sequences used in this study are listed in Table 1.

**TABLE 1:** Primers and sequences used in this study.

Gene	Forward sequence	Reverse sequence
*ALP*	TGA​CCG​TCC​TGC​TGG​AAC​TCG	CCA​CTG​CCA​CAC​TTG​TCA​CAG​AG
*BSP*	AGA​AAG​AGC​AGC​ACG​GTT​GAG​T	GAC​CCT​CGT​AGC​CTT​CAT​AGC​C
*BMP-2*	CAG​CGG​AAG​CGT​CTT​AAG​TCC​AG	GGC​ATG​GTT​GGT​GGA​GTT​CAG​G
*Col-1*	TGT​TGG​TCC​TGC​TGG​CAA​GAA​TG	GTC​ACC​TTG​TTC​GCC​TGT​CTC​AC
*Runx2*	AAC​AGC​AGC​AGC​AGC​AGC​AG	GCA​CGG​AGC​ACA​GGA​AGT​TGG
*SATB2*	GCT​GCT​CAA​AGA​AAT​GAA​CCA​GA	AAA​CTC​CTG​GCA​CTT​GGT​TG
*P53*	TGC​TAG​TCC​CTT​CAC​TGC​CTT​T	AGA​GAC​CCA​GCA​ACT​ACC​AAC​C
*P21*	AGT​GCC​TTG​ACG​ATA​CAG​CTA	TTG​CAC​TGT​ACT​CCT​CTT​GAC​C
*P16*	TTC​ACC​AAA​CGC​CCC​GAA​C	TTC​GAA​TCT​GCA​CCA​TAG​GAG​A
*β-actin*	GTA​AAG​ACC​TCT​ATG​CCA​ACA	GGA​CTC​ATC​GTA​CTC​CTG​CT

#### 2.4.5 Immunofluorescence staining

Expressions of osteogenic and senescence-associated proteins of Y-BMSCs and O-BMSCs cultured with/without OI-exo was detected *via* immunofluorescence staining. Briefly, cells were seeded in 6-well plates at a density of 1*10^5^ cells/well with/without addition of OI-exo. Osteogenic related proteins BMP-2, Col-1 and OSX, as well as senescent related protein SATB2 were detected by immunofluorescence staining. After cultureing for 3 days, the cells were fixed with 4% paraformaldehyde, permeabilized with 0.1% Triton X-100, blocked with 5% BSA and incubated with primary antibodies (Abcam, United Kingdom) overnight. Afterwards, the samples were stained with fluorescent dye-conjugated secondary antibodies (Abcam, United Kingdom) and counter-stained with FITC-phalloidin and DAPI. Images of the immunofluorescence staining were taken by confocal laser-scanning microscopy (CLSM; Leica, Germany).

### 2.5 *In vivo* bone regeneration

#### 2.5.1 Surgical procedure

Preparation of the exosome-loaded scaffolds and rat calvarial defect model were previously described (A. Liu et al., 2021). 8-week young rats and 18-month aged rats were used for calvarial defect model establishment in this study. Briefly, after anesthesia *via* intraperitoneal injection of pentobarbital (3.5 mg/100 g), a 1.0- to 1.5-cm sagittal incision was made on the scalp to expose the calvarium. Trephine bur (Fine Science Tools, United States) was used to made bilateral critical-sized defects (5-mm diameter) on the skull of young or old rats. Different groups of scaffolds were filled into the bone defects. In this study, three experimental groups were set as (*n* = 5): 1) MBG scaffold in old rats (O-MBG); 2) OI-exo-loaded MBG scaffold in old rat (O-exo-MBG); 3) OI-exo-loaded MBG scaffold in young rat (Y-exo-MBG). 12 weeks post-implantation, all rats were anesthetized, and the calvarium specimens were harvested and fixed with 4% paraformaldehyde for further investigation.

#### 2.5.2 Micro-CT

The fixed specimens were scanned using micro-CT system (PerkinElmer Quantum GX, United States) to assess the new bone formation (*n* = 6). The scanning parameters were as follows: 25 mm field of view (FOV) and 50 μm voxel size, 90 KV voltage and 88 μA current. 3D reconstructions were conducted, and the percentage of bone volume to tissue volume (BV/TV) were quantitatively calculated to evaluate new bone formation of different groups.

#### 2.5.3 Sequential fluorescent labeling and VG staining

25 mg/kg tetracycline (TE), 30 mg/kg alizarin red (AR) and 20 mg/kg calcein (CA) were injected at week 3, 6 and 9 respectively, and the specimens (*n* = 3) were obtained at week 12. The specimens were fixed in 4% paraformaldehyde, dehydrated with gradient ethanol, and embedded in Technovit^®^ 7200 VLC resin (Kultzer & Co., Wehrhein, Germany) according to the manufacturer’s instruction. Then the specimens were cut into ∼150 μm thick slices, ground and polished to get final slices ∼40 μm in thickness. The fluorescene of the slices were observed under CLSM (Leica, Germany). Fluorescence-labeled areas of the defect site regions were quantified to reflect the formation of new bone during the correspvan Gieson’sonding periods. The rate of new bone mineralization between two time points was determined by measuring the distance between two corresponding fluorescent stripes using ImageJ. The graphic fluorescent areas were analyzed by Integrated Density/Area using ImageJ.

After observation, staining was performed, and the stained slices were observed using an inverted optical microscope (Leica DMI6000B, Germany).

#### 2.5.4 Histology staining

Samples (*n* = 3) were decalcified using EDTA decalcifying fluid (Boster, China), dehydrated in a graded series of alcohol and embedded in paraffin. 5 μm thick sections were sliced, deparaffined and stained with Hematoxylin/eosin (HE) and Masson’s trichrome agent. The sections were observed using an inverted optical microscope (Leica DMI6000B, Germany).

### 2.6 Statistics analysis

Results were expressed as mean ± standard deviations. All data were generated using at least three independent experiments. Statistical analysis was conducted using one-way analysis of variance (ANOVA). A value of *p* < 0.05 was considered statistically significant.

## 3 Results

### 3.1 Senescent O-BMSCs exhibited reduced proliferation and osteogenicity

**FIGURE 1:**
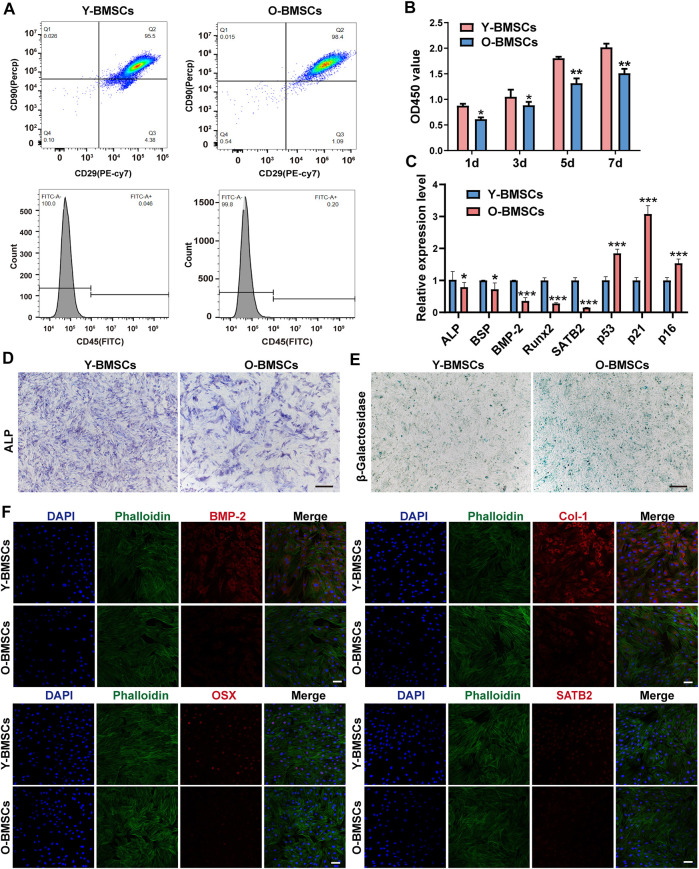
Comparison of the inherent functions of Y-BMSCs and O-BMSCs. **(A)** Flow cytometric profiles of stemness markers CD29/CD45/CD90 for BMSC identification. **(B)** Proliferation by CCK8 assay. **(C)** Osteogenic- and senescence-related gene expressions by rt-qPCR. **(D)** ALP staining. **(E)** SA-β-gal staining. **(F)** Osteogenic- and senescence-related protein levels by immunofluorescence staining. (*p < 0.05, **p < 0.01, ***p < 0.001. Scale bar: 100 μm).

Y-BMSCs and O-BMSCs extracted from 4-week and 18-month rats respectively were identified by flow cytometry (Figure 1A), showing ∼95.5% CD29^+^/CD45^–^/CD90^+^ stem cells in Y-BMSCs and ∼98.4% in O-BMSCs with no significant difference. The result confirmed the reliability of the cells to meet the requirements of subsequent experiments.

The capacity of proliferation, osteogenic differentiation and the level of senescence were compared between Y-BMSCs and O-BMSCs (Figure 1). The CCK-8 assay revealed a statistically significant higher OD450 value of Y-BMSCs than O-BMSCs at all time points (Figure 1B), indicating a reduced proliferation capacity of O-BMSCs. The expression of osteogenic related genes *ALP*, *BSP*, *BMP-2* and *Runx2*, as well as a senescence associated gene *SATB2* significantly decreased in O-BMSCs compared to Y-BMSCs (Figure 1C). Notably, SATB2 might play a potential role in regulating stemness, autophagy and anti-aging properties of BMSCs (Wu et al., 2018). In contrast, the expression of p53, p21 and p16, which were genes positive-related to aging, were upregulated in O-BMSCs (Figure 1C).

The osteogenicity and level of senescence of BMSCs were further visualized *via* ALP and SA-β-gal staining (Figure 1D). O-BMSCs exhibited an evidently reduced ALP activity compared to Y-BMSCs, whereas a significantly increased SA-β-gal activity indicating high level of senescence (Figure 1E). Similarly, the immunofluorescence staining displayed that the expression of osteogenic proteins BMP-2, Col-1 and OSX, as well as aging-related protein SATB2 were significantly decreased in O-BMSCs(Figure 1F), which were consistent with the results of qRT-PCR, ALP and SA-β-gal staining. BMP-2, Col-1 and OSX are important indicators of osteogenic differentiation of BMSCs. Notably, SATB2 is one of senescence-related protein, which is low expressed in O-BMSCs. Altogether, these results demonstrated that, though both were identified stem cells, O-BMSCs exhibited significantly decreased osteogenic differentiation capacity and higher level of senescence than Y-BMSCs.

### 3.2 Osteoinductive exosomes (OI-exos) exerted compromised effect on O-BMSCs

**FIGURE 2:**
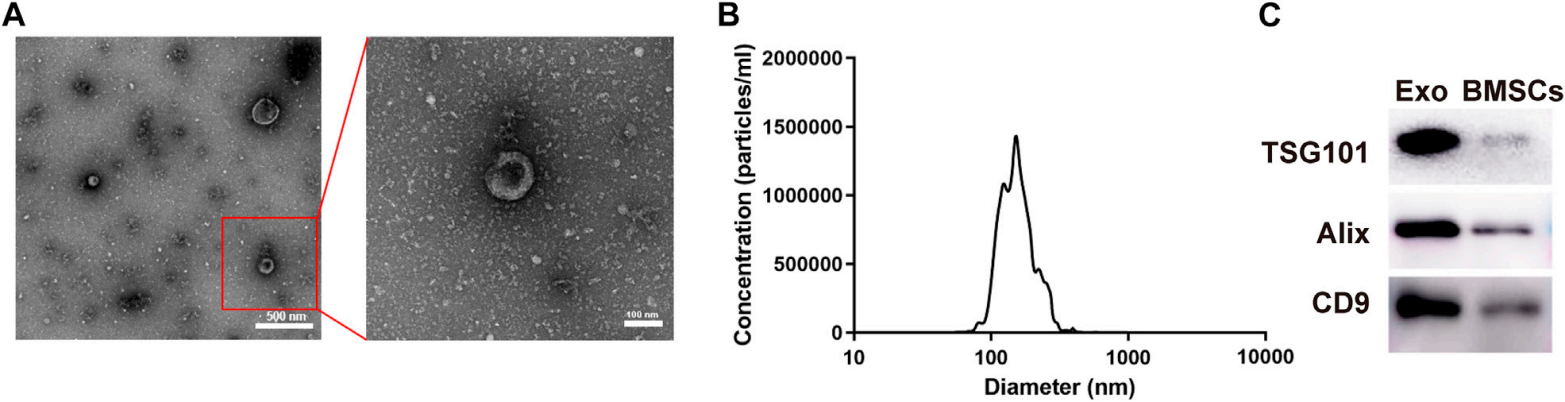
Characterization of the osteoinductive exosome (OI-exo). **(A)** TEM images. **(B)** Particle size distribution measured by NTA. **(C)** Western Blot identification of exosomal markers TSG101, Alix and CD9 in OI-exos and BMSC lysates.

The OI-exos derived from Y-BMSCs could efficiently enhance proliferation and induce osteogenic differentiation of recipient BMSCs (non-senescent type) and enhance bone regeneration in a non-senescent calvarial defect model as reported in our previous study (A. Liu et al., 2021). Herein, OI-exos exhibited a typical round, membrane-bound vesicle appearance with diameters of 50–150 nm as observed in TEM (Figure 2A). The size distribution of OI-exos measured by NTA displayed monodispersity with the peak at ∼150 nm (Figure 2B). Western blotting results identified the exosome-specific markers TSG101, CD9 and Alix confirming the successful isolation of OI-exo (Figure 2C).

**FIGURE 3:**
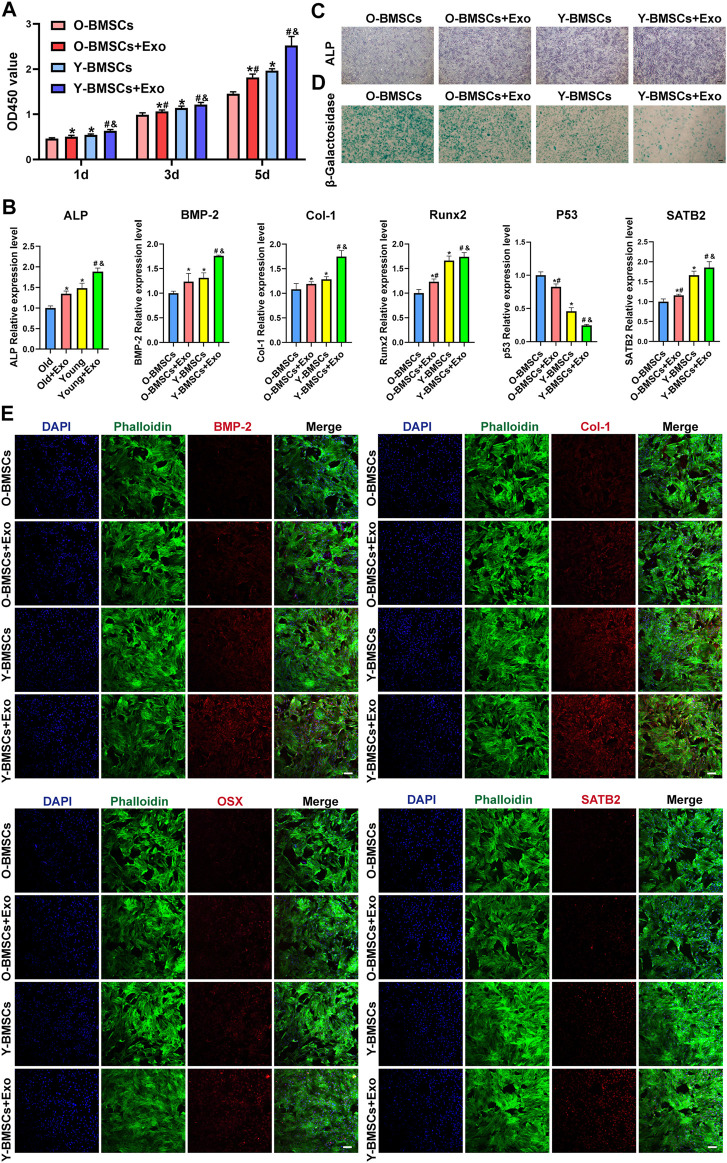
Comparison of the exosomal osteogenic effects on Y-BMSCs and O-BMSCs. **(A)** Proliferation by CCK8 assay. **(B)** ALP staining. **(C)** SA-β-gal staining. **(D)** Osteogenic- and senescence-related gene expressions by RT-qPCR. **(E)** Osteogenic- and senescence-related protein levels by immunofluorescence staining. (* indicates significant differences compared with O-BMSC group; # indicates significant differences compared Y-BMSC group; & indicates significant differences compared the Old + Exo group; *p* < 0.05. Scale bar: 100 μm).

Treatment with OI-exos significantly increased the proliferation of both Y-BMSCs and O-BMSCs (Figure 3A), and enhanced osteogenic related gene expression of both cells (Figure 3B). However, due to the inherent impaired metabolic activity of O-BMSCs, treatment with OI-exos raised partial osteogenic gene expressions (*ALP*, *BMP-2* and *Col-1*) of O-BMSCs to a statistically comparable level to that of untreated Y-BMSCs, whereas proliferation rate, *Runx2* expression and aging-related gene expression (*p53* and *SATB2*) still exhibited a significant difference compared to untreated Y-BMSCs. These data indicated that, instead of rejuvenation, OI-exos could only partially restore the osteogenic function of the senescent O-BMSCs. Moreover, compared to the prominent enhancement in OI-exo-treated Y-BMSCs, O-BMSCs treated with OI-exos exhibited relatively limited improvement of cellular functions. ALP and SA-β-gal staining intuitively presented the significant difference of OI-exo-treated O-BMSCs, though still inferior to the untreated Y-BMSCs (Figures 3C,D). Immunofluorescence staining revealed a similar trend that OI-exos partially restored the osteogenic function of O-BMSCs and reduced the senescent level to a limited extent (Figure 3E).

The above results indicated a compromised effect of OI-exos on O-BMSCs compared to Y-BMSCs, however, the underlying mechanism of which remained further exploration.

### 3.3 *In vivo* bone regeneration in aging rats showed limited enhancement by OI-exo

**FIGURE 4:**
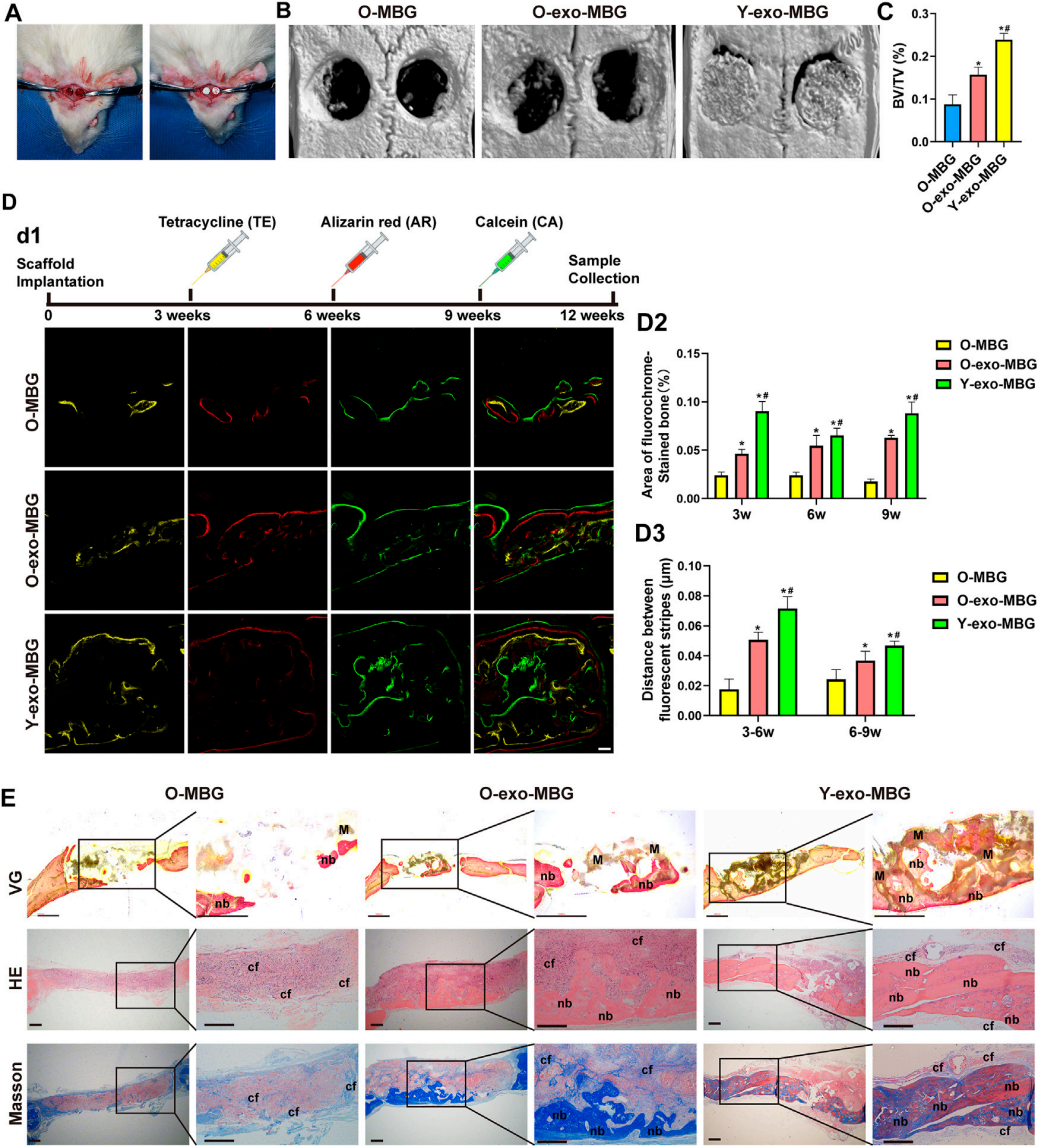
*In vivo* bone regenerative efficacies of OI-exo-loaded scaffolds in young and aged rats. **(A)** Establishment of rat calvarial defect model. **(B)** Micro-CT analysis of bone formation at the defect sites and its **(C)** quantification of bone volume/total volume (BV/TV). **(D)** Sequential fluorescent labeling: d1) fluorescence observation by CLSM, its d2) quantification of stained bone area and d3) distance between stripes. **(E)** Histological evaluation of the bone formation stained with VG (undecalcified), HE and Masson’s trichrome staining. (* indicates significant differences compared with the O-MBG group; # indicates significant differences compared the O-Exo-MBG group; *p* < 0.05. M: material; nb: new bone; cf: collagen fibers).

The *in vivo* bone regenerative efficacies of OI-exo in young and aged individuals were compared using a calvarial defect model (Figure 4A). Hierarchical macro-/micro-/meso-porous MBG scaffolds with inherently osteoinductive ability and structural bioactivity maintenance was applied as a carrier for lyophilized exosomes (exo-MBG) and exerted promising results as reported in our previous study (A. Liu et al., 2021) and reconfirmed in this study (Figure 4). At the end of the experiment time period, the cranial defect site of young rats were occupied by a fusion of residual scaffold material and newly formed bone tissues (Figure 4B), which might be further remodeled into fully regenerated bone by continuous degradation of the residual material by osteoclasts and remineralization of osteoblast. Unexpectedly, the MBG or exo-MBG scaffolds were mostly degraded in old rats by 12 weeks post-implantation as visually presented in micro-CT reconstructed images (Figure 4B). Such accelerated degradation might be related with pro-inflammatory factors that increased with age (Rashad et al., 2019), and the vigorous activity of osteoclasts (which were derived from macrophage, one of the most common immunocytes) might be responsible for the rapid degradation of scaffolds (Almeida et al., 2020).

Highest bone formation (BV/TV) was observed in Y-exo-MBG group, whereas O-MBG and O-exo-MBG exhibited significantly decreased bone volume (Figure 4C). The inferior bone formation in old rats were probably attributed to the excessive inflammatory microenvironment that prematurely degraded the scaffold, which, in normal physiological conditions, should serve as templates for bone remodeling. Non-etheless, O-exo-MBG group displayed significant higher BV/TV than O-MBG group (Figure 4C), indicating the *in vivo* osteoinductive effect of OI-exo. Though with the osteogenic efficacy of OI-exo, the excessive inflammation of senescent individuals that compromised the therapeutic effects of drugs including but not limited to exosomes must be solved *via* further investigation.

Sequential fluorescent labeling of different calcium-binding dyes at different time points revealed the amount and rate of new bone mineralization during the periods of bone regeneration (Figure 4D). At an early stage (week 3), a relatively small amount of bone formation was observed in O-MBG group, which was increased in O-exo-MBG group. Due to the prematured scaffold degradation in old rats, the early-stage bone formation laid the foundation for subsequent bone repair and remodeling, resulting a similar trend in the following time points. Y-exo-MBG group exhibited the highest level of bone mineralization during the whole period. The quantification of bone deposition amount and rate of the three groups were Y-exo-MBG > O-exo-MBG > O-MBG. For each group, stripe distances at early stage (3–6 w) were larger than later stage (6–9 w), suggesting that the new bone formation tendency decreased with time, emphasizing the importance of motivating early-stage bone regeneration.

Histological observation reconfirmed the results of micro-CT and sequential fluorescent labeling (Figure 4E). Van Gieson’s (VG) staining, as the widely acknowledged method for identifying collagen fibers in bone tissue, exhibited the fusion of significantly highest new bone formation in the scaffold porosities in Y-exo-MBG group, and more bone formation in O-exo-MBG group than O-MBG group. Similar results could be observed in H&E and Masson’s trichrome staining images, where tremendous immunocytes were observed in the calvarial defect sites of O-MBG group. In O-exo-MBG group, fibrous connective tissue occupied the non-osseous areas of O-exo-MBG group which might be further remineralized, and the newly formed bone bridged in the bottom of the calvarial defect. In Y-exo-MBG group, massive new bone formation was observed in the macroporosity of the scaffold. The amount of new bone as observed in the histological images were Y-exo-MBG > O-Exo-MBG > O-MBG.

These results demonstrated the positive therapeutic effects of OI-Exo that initiated bone regeneration at an early-stage and resulted in improved bone regenerative outcome, which were attribute to the multi-components of osteogenic-related cargoes in OI-Exo as reported in our previous study (A. Liu et al., 2021). However, the *in vivo* results were consistent with the trend *in vitro*, as the OI-exos could only partially restore the osteogenic function of aged rats, and the underlying mechanism of such compromised exosomal effects needed to be further explored.

### 3.4 Macropinocytosis-involving endocytoses potentially compromised exosomal efficacy in O-BMSCs

**FIGURE 5:**
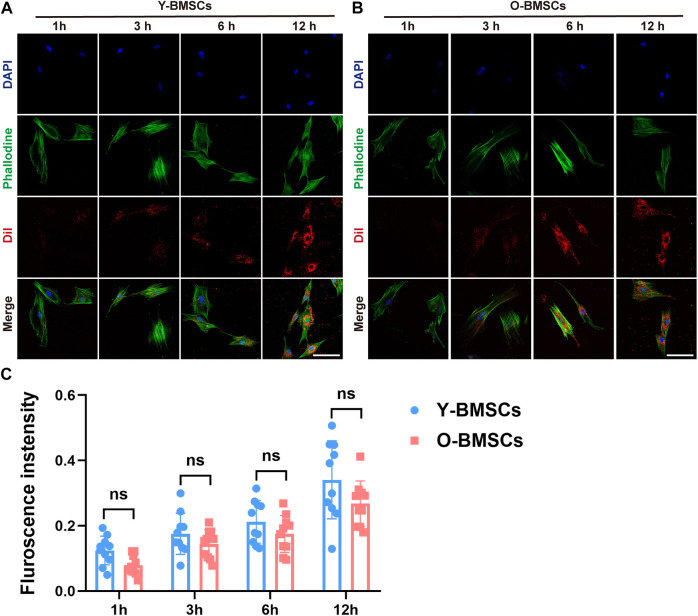
The time-dependent exosome uptake. Fluorescent observation of DiI-labeled exosomes endocytosed by **(A)** Y-BMSCs and **(B)** O-BMSCs at 1h, 3h, 6h and 12 h. **(C)** Quantification of the fluorescent intensity of internalized exosomes. (ns: no significance. Scale bar: 100 μm).

To explore the underlying mechanism of the compromised effects of OI-exos on older rats and older BMSCs, the exosomal uptake pathways of O-BMSCs and Y-BMSCs were compared. DiI-labeled OI-exos were incubated with Y-BMSCs and O-BMSCs for1h, 3h, 6h and 12 h to observe the time-dependent exosome uptake (Figure 5). The red fluorescence in Figures 5A,B revealed the distribution of internalized exosomes in the cytoplasm of Y-BMSCs and O-BMSCs, respectively. The exosomal uptake of both BMSCs exhibited a time-dependent manner, the uptake gradually increases with time and approached a saturation point at 6 h (the fluorescence intensity of 12 h exhibited no statistically significant difference compared to 6 h). The quantification of exosome internalization showed no significant difference between Y-BMSCs and O-BMSCs at each time point (Figure 5C). In both BMSCs, distribution of the internalized exosomes exhibited a relocation tendency toward the cell nuclear after uptake saturation. The results indicated similar uptake capacities of exosomes into Y-BMSCs and O-BMSCs, and explained that OI-exos exerted osteoinductive effects on both young and old cells and individuals. It also excluded the possibility that the compromised effects of OI-exos on O-BMSCs came from different amount of internalization.

**FIGURE 6:**
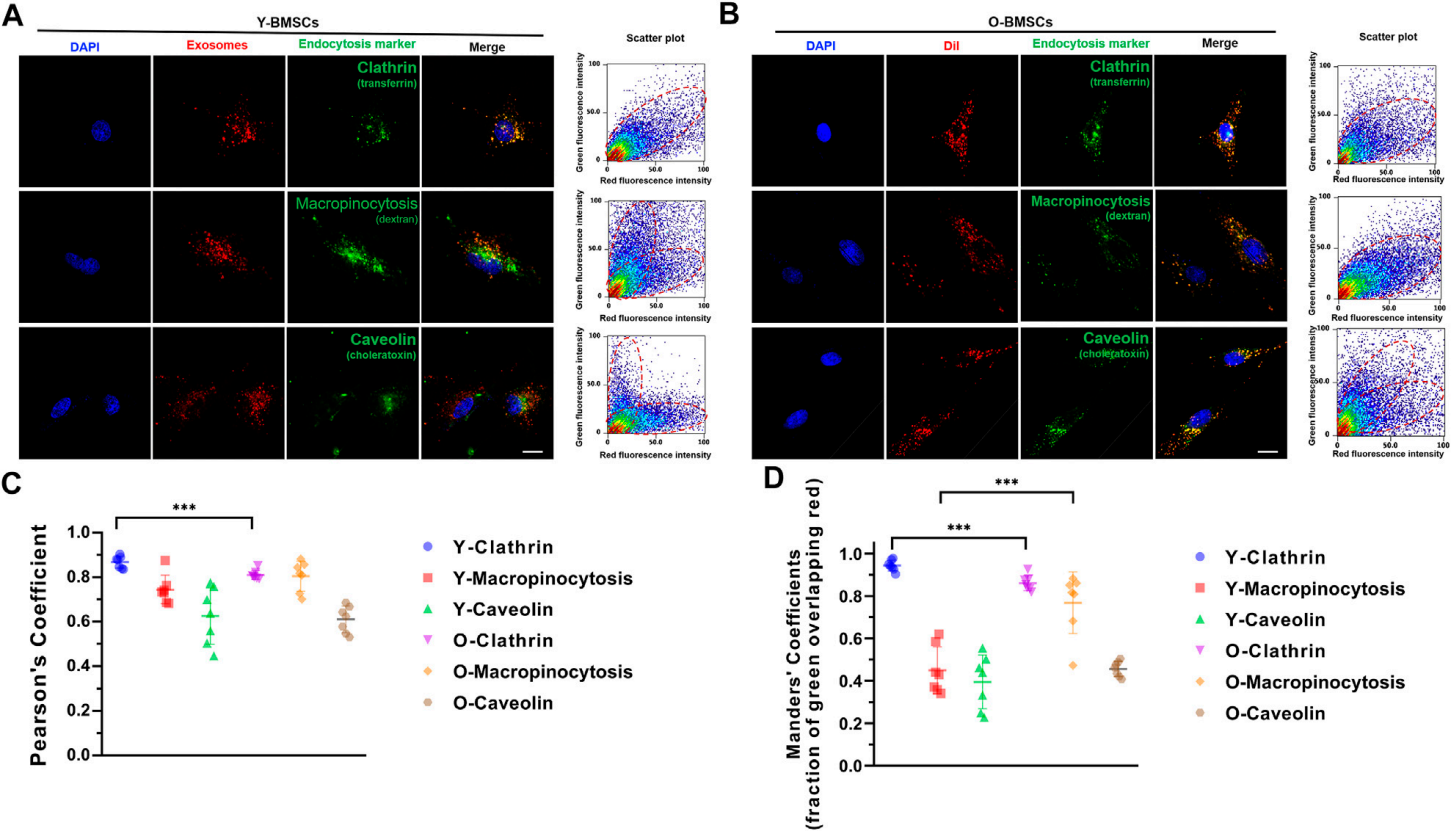
Uptake channels of OI-exos by co-localization of exosomal fluorescence (red) and different endocytic markers (green) in **(A)** Y-BMSCs and **(B)** O-BMSCs. **(C)** Pearson’s confficient analysis and **(D)** Manders’ confficient analysis of colocalization of OI-Exo and endocytic markers. (***p < 0.005. Scale bar: 100 μm).

Further investigation on the uptake channels of OI-exos into the BMSCs were conducted *via* co-localization of the exosomal fluorescence (red) and different endocytic markers (green) (Figure 6). An incubation time of 3 h was selected for a medium internalization amount and avoidance of exosomal relocation at later timepoints. Fluorescence-labeled transferrin, dextran and choleratoxin were applied as markers for clathrin-mediated endocytosis, macropinocytosis, and caveolae-mediated endocytosis, respectively. As shown in Figure 6A, in Y-BMSCs, high degree of co-localization of exosomes and pathway markers was mainly observed in transferrin, but not in dextran and choleratoxin, indicating that the dominant exosomal uptake pathway of Y-BMSCs was *via* clathrin-mediated endocytosis. In contrast, in O-BMSCs, though the highest degree of co-localization was observed in transferrin (Figure 6B), choleratoxin also exhibited a significantly higher Manders’ confficient compared to Y-BMSCs, indicating a potential role of macropinocytosis in exosomal uptake of O-BMSCs (Figure 6D). Pearson’s confficient analyses indicated a significant difference in the co-localization degree of clathrin-mediated endocytosis between Y-BMSCs and O-BMSCs (Figure 6C), whereas both clathrin-mediated endocytosis and macropinocytosis exhibited statistical differences in Manders’ confficient between Y-BMSCs and O-BMSCss (Figure 6D).

**FIGURE 7:**
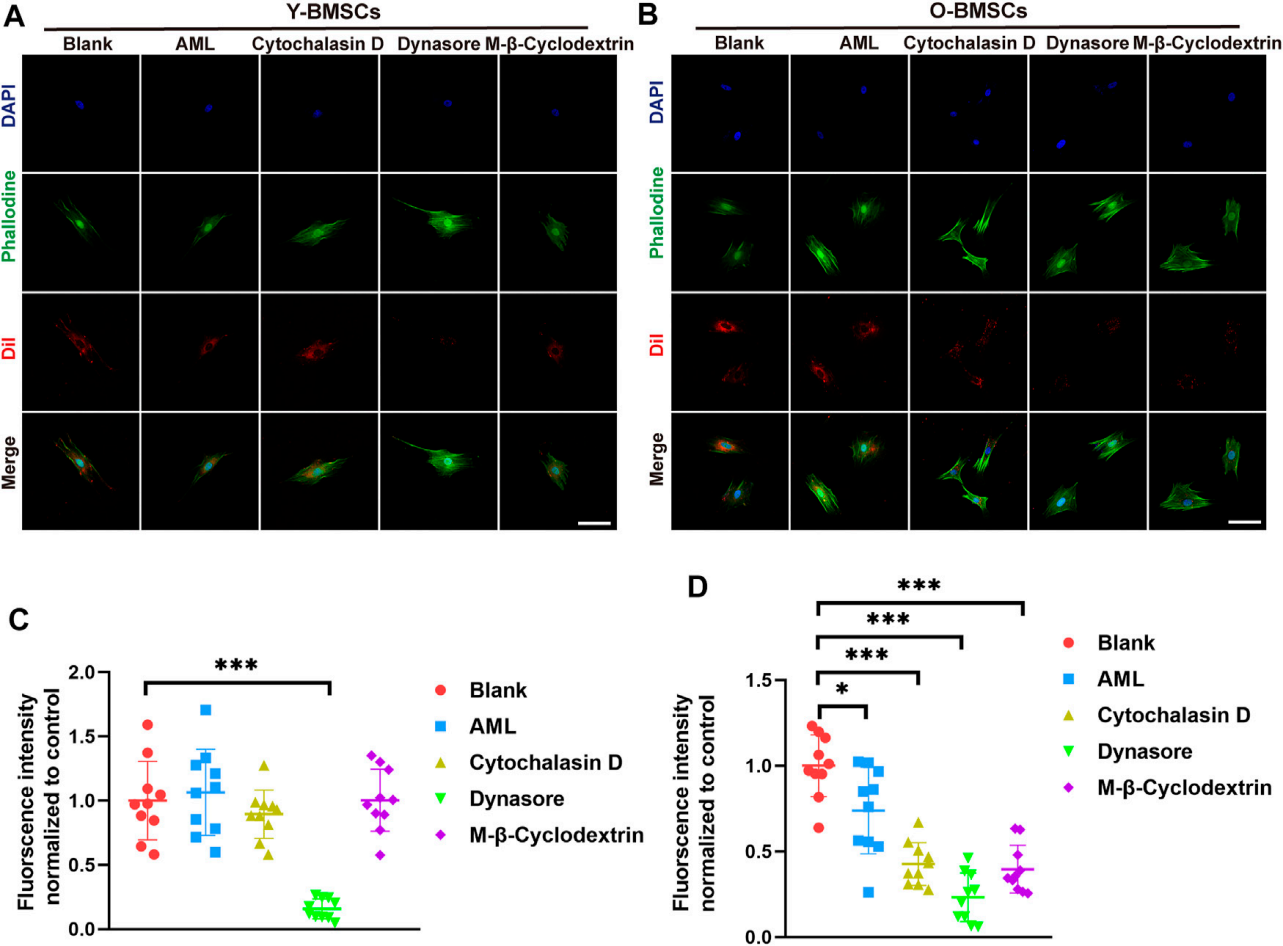
Exosome uptake of **(A)** Y-BMSCs and **(B)** O-BMSCs pre-treated with endocytic pathway inhibitors. Quantification of the fluorescent intensity of internalized exosomes in **(C)** Y-BMSCs and **(D)** O-BMSCs. (*p < 0.05, **p < 0.01, ***p < 0.001. Scale bar: 100 μm).

As co-localization analyses only suggested the potential possibilities, further verification of the endocytic channel were carried out using pathway inhibitors. The exosome uptake of different inhibitor-pretreated BMSCs were quantified to verify the degree of involvement of the corresponding pathway. As shown in Figure 7A, OI-exo uptake by Y-BMSCs was significantly inhibited only by dynasore, an inhibitor of clathrin-mediated endocytosis, indicating that Y-BMSCs internalized exosomes exclusively *via* clathrin-mediated endocytosis. In contrast, though dynasore treatment also showed the highest reduction in uptake, all channel inhibitors exerted significant negative effects on exosome internalization by O-BMSCs (Figure 7B). These results revealed that under a normal physiological cellular environment of non-senescent BMSCs, exosomes were internalized exclusively *via* clathrin-mediated endocytosis (Figure 7C), while senescent BMSCs required additional involvement of macropinocytosis and caveolae-mediated endocytosis to mediate the internalization of exosomes at an equal amount (Figure 7D). This discrepancy in exosomal uptake pathways of young and old cells might be the main direct reason of the compromised exosomal effects.

## 4 Discussion

Endocytic mechanisms control the lipid and protein composition of the plasma membrane, thereby regulating how cells interact with their environments (Svensson et al., 2013; Fan et al., 2019). Different endocytic uptake routes could generate distinct downstream functions (Zuhorn, Kalicharan, and Hoekstra 2002; Mao et al., 2021). Exosomes, as the most common membrane-bound nanovesicles of lipids, proteins, and nucleic acids (Baglio et al., 2015), were internalized as a constitutive phenomenon involved in both physiological and pathological processes (Zhao. X et al., 2022). Although much is known about the exosomal cargoes, the specific mechanisms by which these cargoes are recruited and internalized are less clear. These distinct pathways create endosomal compartments with distinct lumina and surfaces to allow the differential modulation of intracellular events, including the possibility of delivering cargoes to distinct intracellular destinations (Shelke et al., 2019; Arbo et al., 2020).

It is acknowledged that endocytosis intimately regulates many cellular processes including nutrient uptake, cell adhesion and migration, signaling, pathogen entry, synaptic transmission, receptor downregulation, antigen presentation, cell polarity, mitosis, growth and differentiation, and drug delivery (B.Y. Xu et al., 2020). In the present study, OI-exos were internalizationed into Y-BMSCs and O-BMSCs *via* different endocytic routes that may activate different downstream signaling cascades and related proteins, which may be the reason for different efficiencies in promoting bone regeneration.

As non-senescent cellular environment of BMSCs internalized exosomes exclusively *via* clathrin-mediated endocytosis, senescent BMSCs required additional involvement of macropinocytosis and caveolae-mediated endocytosis to mediate the internalization of exosomes. Clathrin-mediated endocytosis was reported as the common endocytic channel that play an important role in the positive regulation of many intracellular signaling cascades (Hoban et al., 2020; Johnson et al., 2021). Caveolae was the most commonly reported non-clathrin coated plasma membrane buds with flask-shaped invaginations that existed on the surface of many mammalian cell types including smooth muscle, type I pneumocytes, fibroblasts, adipocytes, and endothelial cells (Nanbo et al., 2013). It is been reported that exosomes could be internalized by DPSCs and HMSCs *via* the caveolar endocytic mechanism and trigger the P38 mitogen activated protein kinase (MAPK) pathway (Huang et al., 2016). It is speculated that the reduced metabolic activity of senescent BMSCs might also undermine the efficiency of clathrin-mediated endocytosis, thereby evoke the involvement of other endocytic pathways. Macropinocytosis was reported to promote particle uptake. Macropinocytoses of exosomes were also dependent on Na^+^ and PI3K, and could be blocked with the inhibitors of Na^+^-H^+^ ion exchange and PI3K activity, EIPA and LY294002, respectively, which is consistent with our study (Tian et al., 2014).

Macropinocytosis might be involved in some signaling pathways that would negatively affect the osteogenic differentiation in O-BMSCs. Internalization *via* macropinocytosis usually occured from highly ruffled regions of the plasma membrane (Y.X. Li and Pang 2021), and such vigorous plasma membrane activity might be hijacked by pathogens for infectious cell entry (Mercer and Helenius 2009). Hyperstimulation of macropinocytosis by active RAS expression or some medicines can cause cell death, also known as methuosis (Qiu et al., 2022). In some cases, macropinocytosis allowed cancer cells to develop resistance to antitumor drug therapies and reduced the drug efficiencies (Stow, Hung, and Wall 2020). At *Drosophila* neuromuscular junction, the presynaptic Rac1-SCAR pathway mediated BMP-induced receptor macropinocytosis to inhibit BMP growth signaling (Park et al., 2022). Immunity and inflammation shared a common platform with metabolism and cell survival in macropinocytosis, and its non-selective, bulk intake of fluid and membrane resulted in more voluminous and more complex than any other endocytic compartments, its detailed mechanisms remained unclear.

## 5 Conclusion

In this study, a compromised osteogenic effect of therapeutical osteoinductive exosomes on senescent BMSCs/individuals compared with young BMSCs/individuals was discovered. It was demonstrated that non-senescent BMSCs internalized exosomes exclusively *via* clathrin-mediated endocytosis, while senescent BMSCs additionally evoked macropinocytosis and caveolae-mediated endocytosis to mediate the internalization of exosomes. The alteration of endocytic manner of senescent BMSCs might be responsible for the compromised effects of therapeutical exosomes. The phenomena discovered in this study could also be extended to other scenarios where drugs or treatments exerted compromised effects in aging individuals. The influence of endocytic manner, avoidance of macropinocytosis-related negative effects should be taken into considerations in future therapeutic design for aging populations.

## Data Availability

The original contributions presented in the study are included in the article/supplementary material, further inquiries can be directed to the corresponding authors.

## Abbreviations

AR, alizarin red; BV/TV, bone volume to tissue volume; BMSCs, bone marrow stem cells; CA, calcein; CLSM, confocal laser-scanning microscopy; CCK-8, cell counting kit-8 assay; HE, Hematoxylin/eosin; OI-exo, osteoinductive exosome; MBG, mesoporous bioactive glass; FBS, fetal bovine serum; TEM, transmission electron microscope; NTA, nanoparticle tracking analysis; SA-β-Gal, senescence associated *ß*-galactosidase; qRT-PCR, quantitative real-time polymerase chain reaction; FOV, field of view; TE, tetracycline; VG, van Gieson’s.

## References

[B1] Abels E. R. Breakefield X. O. (2016). Introduction to extracellular vesicles: Biogenesis, RNA cargo selection, content, release, and uptake. Cell Mol. Neurobiol. 36 (3), 301–312. 10.1007/s10571-016-0366-z 27053351 PMC5546313

[B2] Almeida A. R. Bessa-Gonçalves M. Vasconcelos D. M. Barbosa M. A. Santos S. G. (2020). Osteoclasts degrade fibrinogen scaffolds and induce mesenchymal stem/stromal osteogenic differentiation. J. Biomed. Mater Res. A 108 (4), 851–862. 10.1002/jbm.a.36863 31845492

[B3] Arbo B. D. Cechinel L. R. Palazzo R. P. Siqueira I. R. (2020). Endosomal dysfunction impacts extracellular vesicle release: Central role in Aβ pathology. Ageing Res. Rev. 58, 101006. 10.1016/j.arr.2019.101006 31891813

[B4] Baglio S. R. Rooijers K. Koppers-Lalic D. Verweij F. J. Pérez Lanzón M. Zini N. (2015). Human bone marrow- and adipose-mesenchymal stem cells secrete exosomes enriched in distinctive miRNA and tRNA species. Stem Cell Res. Ther. 6 (1), 127. 10.1186/s13287-015-0116-z 26129847 PMC4529699

[B5] Cai G. P. Liu Y. L. Luo L. P. Xiao Y. Jiang T. J. Yuan J. (2022). Alkbh1-mediated DNA N6-methyladenine modification regulates bone marrow mesenchymal stem cell fate during skeletal aging. Cell Prolif. 55 (2), e13178. 10.1111/cpr.13178 35018683 PMC8828262

[B6] Cancedda R. Giannoni P. Mastrogiacomo M. (2007). A tissue engineering approach to bone repair in large animal models and in clinical practice. Biomaterials 28 (29), 4240–4250. 10.1016/j.biomaterials.2007.06.023 17644173

[B7] Cao C. Huang Y. Tang Q. Zhang C. Shi L. Zhao J. (2018). Bidirectional juxtacrine ephrinB2/Ephs signaling promotes angiogenesis of ECs and maintains self-renewal of MSCs. Biomaterials 172, 1–13. 10.1016/j.biomaterials.2018.04.042 29709731

[B8] Costa Verdera H. Gitz-Francois J. J. Schiffelers R. M. Vader P. (2017). Cellular uptake of extracellular vesicles is mediated by clathrin-independent endocytosis and macropinocytosis. J. Control Release 266, 100–108. 10.1016/j.jconrel.2017.09.019 28919558

[B9] Fan W. Guo J. Gao B. Zhang W. Ling L. Xu T. (2019). Flotillin-mediated endocytosis and ALIX-syntenin-1-mediated exocytosis protect the cell membrane from damage caused by necroptosis. Sci. Signal 12 (583), eaaw3423. 10.1126/scisignal.aaw3423 31138766

[B10] Gurung S. Perocheau D. Touramanidou L. Baruteau J. (2021). The exosome journey: From biogenesis to uptake and intracellular signalling. Cell Commun. Signal 19 (1), 47. 10.1186/s12964-021-00730-1 33892745 PMC8063428

[B11] Hoban K. Lux S. Y. Poprawski J. Zhang Y. Shepherdson J. Castiñeira P. G. (2020). ESCRT-dependent protein sorting is required for the viability of yeast clathrin-mediated endocytosis mutants. Traffic 21 (6), 430–450. 10.1111/tra.12731 32255230 PMC11376963

[B12] Horibe S. Tanahashi T. Kawauchi S. Murakami Y. Rikitake Y. (2018). Mechanism of recipient cell-dependent differences in exosome uptake. BMC Cancer 18 (1), 47. 10.1186/s12885-017-3958-1 29306323 PMC5756423

[B13] Hu L. Xie X. Xue H. Wang T. Panayi A. C. Lin Z. (2022). MiR-1224-5p modulates osteogenesis by coordinating osteoblast/osteoclast differentiation via the Rap1 signaling target ADCY2. Exp. Mol. Med. 54 (7), 961–972. 10.1038/s12276-022-00799-9 35831436 PMC9355958

[B14] Hu Y. Zhang Y. Ni C. Y. Chen C. Y. Rao S. S. Yin H. (2020). Human umbilical cord mesenchymal stromal cells-derived extracellular vesicles exert potent bone protective effects by CLEC11A-mediated regulation of bone metabolism. Theranostics 10 (5), 2293–2308. 10.7150/thno.39238 32089743 PMC7019162

[B15] Huang C. C. Narayanan R. Alapati S. Ravindran S. (2016). Exosomes as biomimetic tools for stem cell differentiation: Applications in dental pulp tissue regeneration. Biomaterials 111, 103–115. 10.1016/j.biomaterials.2016.09.029 27728810 PMC5293278

[B16] Johnson A. Dahhan D. A. Gnyliukh N. Kaufmann W. A. Zheden V. Costanzo T. (2021). The TPLATE complex mediates membrane bending during plant clathrin-mediated endocytosis. Proc. Natl. Acad. Sci. U. S. A. 118 (51), e2113046118. 10.1073/pnas.2113046118 34907016 PMC8691179

[B17] Joshi B. S. de Beer M. A. Giepmans B. N. G. Zuhorn I. S. (2020). Endocytosis of extracellular vesicles and release of their cargo from endosomes. ACS Nano 14 (4), 4444–4455. 10.1021/acsnano.9b10033 32282185 PMC7199215

[B18] Kang X. Chen L. Yang S. Gong Z. Hu H. Zhang X. (2022). Zuogui Wan slowed senescence of bone marrow mesenchymal stem cells by suppressing Wnt/β-catenin signaling. J. Ethnopharmacol. 294, 115323. 10.1016/j.jep.2022.115323 35483559

[B19] Li C. Wei G. J. Xu L. Rong J. S. Tao S. Q. Wang Y. S. (2017). The involvement of senescence induced by the telomere shortness in the decline of osteogenic differentiation in BMSCs. Eur. Rev. Med. Pharmacol. Sci. 21 (5), 1117–1124. 28338178

[B20] Li G. Zhu Q. Wang B. Luo R. Xiao X. Zhang Y. (2021). Rejuvenation of senescent bone marrow mesenchymal stromal cells by pulsed triboelectric stimulation. Adv. Sci. (Weinh) 8 (18), e2100964. 10.1002/advs.202100964 34258884 PMC8456218

[B21] Li Q. Xu R. Lei K. Yuan Q. (2022). Insights into skeletal stem cells. Bone Res. 10 (1), 61. 10.1038/s41413-022-00235-8 36261411 PMC9581935

[B22] Li X. Wang X. Zhang C. Wang J. Wang S. Hu L. (2022). Dysfunction of metabolic activity of bone marrow mesenchymal stem cells in aged mice. Cell Prolif. 55 (3), e13191. 10.1111/cpr.13191 35088483 PMC8891618

[B23] Li Y. X. Pang H. B. (2021). Macropinocytosis as a cell entry route for peptide-functionalized and bystander nanoparticles. J. Control Release 329, 1222–1230. 10.1016/j.jconrel.2020.10.049 33622520 PMC7905157

[B24] Liao C. M. Luo T. von der Ohe J. de Juan Mora B. Schmitt R. Hass R. (2021). Human MSC-derived exosomes reduce cellular senescence in renal epithelial cells. Int. J. Mol. Sci. 22 (24), 13562. 10.3390/ijms222413562 34948355 PMC8709122

[B25] Liu A. Lin D. Zhao H. Chen L. Cai B. Lin K. (2021). Optimized BMSC-derived osteoinductive exosomes immobilized in hierarchical scaffold via lyophilization for bone repair through Bmpr2/Acvr2b competitive receptor-activated Smad pathway. Biomaterials 272, 120718. 10.1016/j.biomaterials.2021.120718 33838528

[B26] Liu L. Liu Y. Feng C. Chang J. Fu R. Wu T. (2019). Lithium-containing biomaterials stimulate bone marrow stromal cell-derived exosomal miR-130a secretion to promote angiogenesis. Biomaterials 192, 523–536. 10.1016/j.biomaterials.2018.11.007 30529871

[B27] Liu X. Zhang L. Xu Z. Xiong X. Yu Y. Wu H. (2022). A functionalized collagen-I scaffold delivers microRNA 21-loaded exosomes for spinal cord injury repair. Acta Biomater. 154, 385–400. 10.1016/j.actbio.2022.10.027 36270583

[B28] Luo Z. Qi B. Sun Y. Chen Y. Lin J. Qin H. (2022). Engineering bioactive M2 macrophage-polarized, anti-inflammatory, miRNA-based liposomes for functional muscle repair: From exosomal mechanisms to biomaterials. Small 18 (34), e2201957. 10.1002/smll.202201957 35802903

[B29] Mao L. Liao C. Qin J. Gong Y. Zhou Y. Li S. (2021). Phosphorylation of SNX27 by MAPK11/14 links cellular stress-signaling pathways with endocytic recycling. J. Cell Biol. 220 (4), e202010048. 10.1083/jcb.202010048 33605979 PMC7901142

[B30] McKelvey K. J. Powell K. L. Ashton A. W. Morris J. M. McCracken S. A. (2015). Exosomes: Mechanisms of uptake. J. Circ. Biomark. 4, 7. 10.5772/61186 28936243 PMC5572985

[B31] Mercer J. Helenius A. (2009). Virus entry by macropinocytosis. Nat. Cell Biol. 11 (5), 510–520. 10.1038/ncb0509-510 19404330

[B32] Nanbo A. Kawanishi E. Yoshida R. Yoshiyama H. (2013). Exosomes derived from Epstein-Barr virus-infected cells are internalized via caveola-dependent endocytosis and promote phenotypic modulation in target cells. J. Virol. 87 (18), 10334–10347. 10.1128/jvi.01310-13 23864627 PMC3753980

[B33] O'Brien K. Breyne K. Ughetto S. Laurent L. C. Breakefield X. O. (2020). RNA delivery by extracellular vesicles in mammalian cells and its applications. Nat. Rev. Mol. Cell Biol. 21 (10), 585–606. 10.1038/s41580-020-0251-y 32457507 PMC7249041

[B34] Park H. G. Kim Y. D. Cho E. Lu T. Y. Yao C. K. Lee J. (2022). Vav independently regulates synaptic growth and plasticity through distinct actin-based processes. J. Cell Biol. 221 (10), e202203048. 10.1083/jcb.202203048 35976098 PMC9388202

[B35] Paschalis E. P. Fratzl P. Gamsjaeger S. Hassler N. Brozek W. Eriksen E. F. (2016). Aging versus postmenopausal osteoporosis: Bone composition and maturation kinetics at actively-forming trabecular surfaces of female subjects aged 1 to 84 years. J. Bone Min. Res. 31 (2), 347–357. 10.1002/jbmr.2696 26308158

[B36] Peng H. Guo Q. Xiao Y. Su T. Jiang T. J. Guo L. J. (2020). ASPH regulates osteogenic differentiation and cellular senescence of BMSCs. Front. Cell Dev. Biol. 8, 872. 10.3389/fcell.2020.00872 33015050 PMC7494742

[B37] Qiu Z. Liu W. Zhu Q. Ke K. Zhu Q. Jin W. (2022). The role and therapeutic potential of macropinocytosis in cancer. Front. Pharmacol. 13, 919819. 10.3389/fphar.2022.919819 36046825 PMC9421435

[B38] Rashad A. Suliman S. Mustafa M. Pedersen T. Ø. Campodoni E. Sandri M. (2019). Inflammatory responses and tissue reactions to wood-Based nanocellulose scaffolds. Mater Sci. Eng. C Mater Biol. Appl. 97, 208–221. 10.1016/j.msec.2018.11.068 30678905

[B39] Shelke G. V. Yin Y. Jang S. C. Lässer C. Wennmalm S. Hoffmann H. J. (2019). Endosomal signalling via exosome surface TGFβ-1. J. Extracell. Vesicles 8 (1), 1650458. 10.1080/20013078.2019.1650458 31595182 PMC6764367

[B40] Stow J. L. Hung Y. Wall A. A. (2020). Macropinocytosis: Insights from immunology and cancer. Curr. Opin. Cell Biol. 65, 131–140. 10.1016/j.ceb.2020.06.005 32745890

[B41] Svensson K. J. Christianson H. C. Wittrup A. Bourseau-Guilmain E. Lindqvist E. Svensson L. M. (2013). Exosome uptake depends on ERK1/2-heat shock protein 27 signaling and lipid Raft-mediated endocytosis negatively regulated by caveolin-1. J. Biol. Chem. 288 (24), 17713–17724. 10.1074/jbc.M112.445403 23653359 PMC3682571

[B42] Tian T. Zhu Y. L. Zhou Y. Y. Liang G. F. Wang Y. Y. Hu F. H. (2014). Exosome uptake through clathrin-mediated endocytosis and macropinocytosis and mediating miR-21 delivery. J. Biol. Chem. 289 (32), 22258–22267. 10.1074/jbc.M114.588046 24951588 PMC4139237

[B43] Tkach M. Théry C. (2016). Communication by extracellular vesicles: Where we are and where we need to go. Cell 164 (6), 1226–1232. 10.1016/j.cell.2016.01.043 26967288

[B44] Valadi H. Ekström K. Bossios A. Sjöstrand M. Lee J. J. Lötvall J. O. (2007). Exosome-mediated transfer of mRNAs and microRNAs is a novel mechanism of genetic exchange between cells. Nat. Cell Biol. 9 (6), 654–659. 10.1038/ncb1596 17486113

[B45] Wang X. D. Li S. Y. Zhang S. J. Gupta A. Zhang C. P. Wang L. (2020). The neural system regulates bone homeostasis via mesenchymal stem cells: A translational approach. Theranostics 10 (11), 4839–4850. 10.7150/thno.43771 32308753 PMC7163440

[B46] Wu G. Xu R. Zhang P. Xiao T. Fu Y. Zhang Y. (2018). Estrogen regulates stemness and senescence of bone marrow stromal cells to prevent osteoporosis via ERβ-SATB2 pathway. J. Cell Physiol. 233 (5), 4194–4204. 10.1002/jcp.26233 29030963

[B47] Xu B. Y. Tang X. D. Chen J. Wu H. B. Chen W. S. Chen L. (2020). Rifampicin induces clathrin-dependent endocytosis and ubiquitin-proteasome degradation of MRP2 via oxidative stress-activated PKC-ERK/JNK/p38 and PI3K signaling pathways in HepG2 cells. Acta Pharmacol. Sin. 41 (1), 56–64. 10.1038/s41401-019-0266-0 31316180 PMC7468545

[B48] Xu R. Shen X. Si Y. Fu Y. Zhu W. Xiao T. (2018). MicroRNA-31a-5p from aging BMSCs links bone formation and resorption in the aged bone marrow microenvironment. Aging Cell 17 (4), e12794. 10.1111/acel.12794 29896785 PMC6052401

[B49] Yang F. Yang L. Li Y. Yan G. Feng C. Liu T. (2017). Melatonin protects bone marrow mesenchymal stem cells against iron overload-induced aberrant differentiation and senescence. J. Pineal Res. 63 (3), e12422. 10.1111/jpi.12422 28500782

[B50] Yi L. Ju Y. He Y. Yin X. Xu Y. Weng T. (2021). Intraperitoneal injection of Desferal® alleviated the age-related bone loss and senescence of bone marrow stromal cells in rats. Stem Cell Res. Ther. 12 (1), 45. 10.1186/s13287-020-02112-9 33413663 PMC7791659

[B51] Yu B. Wang C. Y. (2016). Osteoporosis: The result of an 'aged' bone microenvironment. Trends Mol. Med. 22 (8), 641–644. 10.1016/j.molmed.2016.06.002 27354328 PMC4969144

[B52] Zhao X. Wang Q. Zhu G. Ma J. Lin N. (2022). Size effect of cellulose nanocrystals in cellular internalization and exosome-packaging exocytosis. Carbohydr. Polym. 298, 120131. 10.1016/j.carbpol.2022.120131 36241332

[B53] Zhao Y. He J. Qiu T. Zhang H. Liao L. Su X. (2022). Epigenetic therapy targeting bone marrow mesenchymal stem cells for age-related bone diseases. Stem Cell Res. Ther. 13 (1), 201. 10.1186/s13287-022-02852-w 35578312 PMC9109405

[B54] Zuhorn I. S. Kalicharan R. Hoekstra D. (2002). Lipoplex-mediated transfection of mammalian cells occurs through the cholesterol-dependent clathrin-mediated pathway of endocytosis. J. Biol. Chem. 277 (20), 18021–18028. 10.1074/jbc.M111257200 11875062

